# Intelligence

**Published:** 1808-01

**Authors:** 


### INTELLIGENCE.

Professor DAVY has lately given to the Royal Society, in reading the Bakerian Lecture, an account of his important discoveries relating to the decomposition or analysis of the Fixed Alkalis. Excepting Galvanism, nothing of greater consequence in chemical science has taken place since the discoveries of Priestley and Cavendish. Mr. Davy, in the Bakerian Lecture of last year, on the agencies of electricity, suggested the probability, that other bodies, not then enumerated, might be decomposed by means of that fluid. Since then by several powerful galvanic troughs, consisting of one hundred pairs of plates of six inches square, and one hundred and fifty pairs of four inches square, he has succeeded in decomposing potash and soda. This was effected by placing moistened potash or soda on a plate of platina, and exposing it to the Galvanic circle. Oxygen was disengaged, and these alkalis were reduced to their primitive base, a peculiar and highly inflammable matter, which assumes the form and appearance of small globules of mercury. The globules are lighter than any other fluid, as they swim in distilled naptha. The base of potash is of a specific gravity as six to ten of water. At the freezing point these globules are hard and brittle, and when broken and examined with a microscope they present a number of facettes with the appearance of crystallizations; at 40° of Fahrenheit they are soft, and can scarcely be distinguished from globules of quicksilver; at 60° they are fluid, and at 100° volatile. When exposed to the atmosphere they rapidly imbibe oxygen, and re-assume their alkaline character. In distilled naptha they may be kept four or five days; but if exposed either to the atmosphere or to oxygen gas, they almost instantly become incrusted with a coat of regenerated alkali; this incrustation can be removed, and the reduced globule will remain in naptha, or separated from all contact with oxygen as before; the naptha forming a thin film round the globule, and excluding the contact of oxygen. In some experiments on these newly discovered bodies, Mr. Davy took one part of the base of alkali and two of mercury, estimated by bulk, or about one part of the base to 48 of mercury by weight, and formed an amalgam, which when applied in the circle of a galvanic battery, which produced intense heat to iron, silver, gold, or platina, immediately dissolved these, and converted them into oxides, in which process alkali was regenerated. Glass, as well as all other metallic bodies, was also dissolved by the application of this substance: the base of the alkali seizing the oxygen of the manganese and of the minium, potash was regenerated. One of these globules placed on a piece of ice, dissolved it, and burnt with a bright flame, giving out an intense heat. Potash was found on the product of the dissolved ice. Nearly the same effects followed when a globule was thrown into water; in both cases a great quantity of hydrogen gas was rapidly liberated. When laid on a piece of moistened turmeric paper, the globule seemed instantly to acquire an intense heat; but so so rapid was its movement in quest of the moisture, that no part of the paper was burnt, only an intense deep red stain marked the course it followed, and showed a reproduction of alkali. The specific gravity of the base of soda is as 7 to 10 of water, and is fixed in a temperature of about 150°, and fluid at 180°.

Mr. Davy next tried its effects on the phosphats, phosphurets, and the greater part of the salts of the first and second degree of oxydizement, all of which it decomposed, seizing their oxygen, and reassuming its alkaline qualities. The specific quality of this amalgam, after a number of experiments, was found by means of a mixture of oil of sassafras with distilled naptha, in which a globule remained either buoyant at top, or quiescent at bottom, in a fluid weighing as nine to ten of water. A great variety of experiments were made to ascertain the difference of the base of *potash* from that of *soda;* and from the medium taken of numerous analytical, and of nine synthetical, experiments, it appeared that 100 parts of potash contain 15 oxygen, and 85 of inflammable base, and that the same quantity of soda contains 20 oxygen, and 80 base. On an examination of the volatile alkali, which chemists led by systematic theory have rather hastily taken for granted that it consists merely of hydrogen and nitrogen, Mr. Davy, after a great number of complex experiments, in which he was assisted by Messrs. Pepys and Allen, ascertained that oxygen is also an essential ingredient in ammonia, 100 grains of the latter yielding 20 of the former; but this result depended too much on eudiometrical calculation to be received as an established fact. Mr. Davy on making some general observations on the series of *new facts* here disclosed, related some miscellaneous experiments on the muriatic and fluoric acids, all of which tended to prove that oxygen is one of their constituent principles. The earths of barytes and strontites, as being most analogous to the alkalis, were likewise examined, and both yielded considerable quantities of oxygen. Mr. Davy concluded by remarking the impropriety of limiting the term oxygen to a specific character, as opposed to that of alkali, observed the necessity of improving the nomenclature in consequence of the *new facts* now discovered, and the influence of this " metallary base as it might be called" on other bodies; and suggested the importance and extent of the new field these facts opened to Geology, as likely to lead to numerous discoveries relative to the formation of various stones, strata, and mountains.

---

Mr. Proust, a physician of some eminence at Paris, has lately endeavoured to prove that the cause of Insanity is seated not so much in the head as in the stomach and bowels. He has observed, that the contents of the bowels, in those who have died while under this disorder, are replete with mucous or bilious matter, more or less discoloured and dark. Worms are often found, and the inner membrane of the bowels is constantly reddish, or even changed altogether from its proper colour in diverse points of its surface. The gall bladder and its ducts are always dilated, and frequently frequently contain concretions. The liver too is enlarged and swelled. These symptoms, Mr. Proust conceives, establish the theory that the seat of the disease is in the stomach and intestines.

---

Mr. Brookes will commence his Spring Course of Lectures on Anatomy, Physiology, and Surgery, on Tuesday, January 19, at two o'clock. Spacious apartments, thoroughly ventilated, and replete with every convenience, are open every morning for the purposes of dissecting and injecting, where Mr. Brookes attends to direct the Students, and demonstrate the various parts as they appear on dissection.

Mr. Taunton's Spring Course of Lectures on Anatomy, Pathology, and Surgery, will commence on Saturday January 23, at eight o'clock in the evening precisely, and be continued every Tuesday, Thursday, and Saturday, at the same hour. Particulars may be had on applying to Mr. Taunton, Greville Street, Hatton Garden.

General Dispensary, Aldersgate Street.—Doctor Clutterbuck, one of the Physicians to this Institution, purposes, about the middle of January, to begin a Course of Lectures on the Theory and Practice of Physic. The Course will include the Physiology, or Doctrine of Functions, and also an Outline of the Materia Medica. The practical part will be illustrated by occasional reference to the practice of the Dispensary Further particulars may be known on application at the Dispensary, or at No. 17, St. Paul's Church Yard.

Mr. Chevalier, Surgeon Extraordinary to the Prince of Wales, and Surgeon to the Westminster General Dispensary, will begin his next Course of Lectures on the Principles and Operations of Surgery, on Monday, the 11th of January, at eight o'clock in the evening, at his house, in South Audley Street, Grosvenor Square, where printed particulars may be had.

Dr. Clarke and Mr. Clarke will begin a Course of their Lectures, on Friday, January 22. The Lectures are read every day, at the Lecture Room, No. 10, Upper John Street, Golden Square, from a quarter past ten o'clock in the morning till a quarter past eleven, for the convenience of students attending the Hospitals. For particulars apply to Dr. Clarke, No. 1, New Burlington Street; or to Mr. Clarke, No. 10, Upper John Street, Golden Square.

Dr. Clough, Physician and Manmidwife to the St. Mary-le-bone General Dispensary, and Endeavour Society, will, on Monday the 11th of January, at ten o'clock in the morning, resume his Course of Lectures on the Theory and Practice of Midwifery, and on the Diseases of Women and Children. An Evening Course, for the convenience of such Students as cannot attend the Morning Lectures, will be given on Tuesdays, Thursdays, and Saturdays, during the winter, at seven. The first Evening Lecture will commence on Tuesday the 12th inst.

Mr.

Mr. Gunning, Surgeon to St. George's Hospital, will commence his Lectures on the Principles and Operations of Surgery, on Friday, the 22d of January, at eight o'clock in the evening. Particulars may be known at Mr. Gunning's house, 45, Conduit Street, Hanover Square, or at St. George's Hospital.

Dr. Reid's Introductory Lecture to his next Course on the Theory and Practice of Medicine, will be given on Wednesday the 13th of January, at eight o'clock in the evening, at his house, in Grenville Street, Brunswick Square; at which place the ensuing Lectures will be delivered at ten o'clock in the morning, on Monday, Wednesday, and Friday, until the conclusion of the Course, which will last three months.

---

### *Diseases and Casualties in London in the Year* 1807.

| | | | | | |
|---|---|---|---|---|---|
| Abortive and Stillborn | 481 | Grief | 10 | St. Vitus's Dance | 1 |
| Abſceſs | 50 | Headmouldſhot, Horſeſhoehead, and Water in the Head | 209 | Swelling | 3 |
| Aged | 1424 | Jaundice | 29 | Teeth | 322 |
| Ague | 1 | Jaw Locked | 5 | Thruſh | 43 |
| Apoplexy & Suddenly | 242 | Impoſthume | 2 | Tumour | 1 |
| Aſthma and Phthiſic | 523 | Inflammation | 632 | Vomiting and Looſeneſs | 3 |
| Bedridden | 2 | Influenza | 0 | Worms | 5 |
| Bile | 3 | Inoculation | 0 | | |
| Bleeding | 22 | Itch | 0 | Bit by Mad Dogs | 2 |
| Burſten and Rupture | 13 | Lethargy | 3 | Broken Limbs | 2 |
| Cancer | 83 | Livergrown | 19 | Broken Neck | 0 |
| Canker | 2 | Lumbago | 0 | Bruiſed | 1 |
| Chicken Pox | 3 | Lunatick | 135 | Burnt | 36 |
| Childbed | 164 | Meaſles | 452 | Choaked | 0 |
| Colds | 10 | Miſcarriage | 8 | Drowned | 111 |
| Colic, Gripes, &c. | 14 | Mortification | 210 | Exceſſive Drinking | 9 |
| Conſumption | 4964 | Palſy | 106 | Executed | 5 |
| Convulſions | 3994 | Palpitation of the Heart | 1 | Found Dead | 13 |
| Cough, and Hooping Cough | 439 | Piles | 0 | Fractured | 1 |
| Cow Pox | 0 | Pleuriſy | 32 | Frighted | 4 |
| Cramp | 0 | Purples | 1 | Killed by Falls and ſeveral other Accidents | 101 |
| Croup | 57 | Quinſy | 4 | Killed by Fighting | 0 |
| Diabetes | 1 | Rheumatiſm | 5 | Killed Themſelves | 45 |
| Dropſy | 790 | Riſing of the Lights | 1 | Murdered | 2 |
| Eaten by Lice | 0 | Scurvy | 0 | Poiſoned | 1 |
| Evil | 4 | Small Pox | 1297 | Scalded | 8 |
| Fevers of all kinds | 1033 | Sore Throat | 4 | Shot | 0 |
| Fiſtula | 3 | Sores and Ulcers | 12 | Smothered | 0 |
| Flux | 8 | St. Anthony's Fire | 3 | Starved | 0 |
| French Pox | 26 | Spaſm | 12 | Strangled | 0 |
| Gout | 32 | Stoppage in the Stom. | 14 | Suffocated | 11 |
| Gravel, Stone, and Strangury | 10 | | | | |

| | | | |
|---|---|---|---|
| Chriſtened | Males | 9812 | In all 19416 |
| | Females | 9604 | |
| Buried | Males | 9269 | In all 18334 |
| | Females | 9038 | |

Whereof have died,

| | | | |
|---|---|---|---|
| Under Two Years of Age | 5443 | Fifty and Sixty | 1665 |
| Between Two and Five | 2010 | Sixty and Seventy | 1507 |
| Five and Ten | 737 | Seventy and Eighty | 1158 |
| Ten and Twenty | 581 | Eighty and Ninety | 462 |
| Twenty and Thirty | 1160 | Ninety and a Hundred | 49 |
| Thirty and Forty | 1883 | A Hundred and One | 1 |
| Forty and Fifty | 1677 | A Hundred and Two | 1 |

Increaſed in the Burials this Year 396.

